# The recipes of *Philosophy of Science*: Characterizing the semantic structure of corpora by means of topic associative rules

**DOI:** 10.1371/journal.pone.0242353

**Published:** 2020-11-18

**Authors:** Christophe Malaterre, Jean-François Chartier, Francis Lareau

**Affiliations:** 1 Département de philosophie, Université du Québec à Montréal (UQAM), Montréal, Québec, Canada; 2 Centre interuniversitaire de recherche sur la science et la technologie (CIRST), Montréal, Québec, Canada; 3 Département d’informatique, Université du Québec à Montréal (UQAM), Montréal, Québec, Canada; University Of Bristol, UNITED KINGDOM

## Abstract

Scientific articles have semantic contents that are usually quite specific to their disciplinary origins. To characterize such semantic contents, topic-modeling algorithms make it possible to identify topics that run throughout corpora. However, they remain limited when it comes to investigating the extent to which topics are jointly used together in specific documents and form particular associative patterns. Here, we propose to characterize such patterns through the identification of “topic associative rules” that describe how topics are associated within given sets of documents. As a case study, we use a corpus from a subfield of the humanities—the philosophy of science—consisting of the complete full-text content of one of its main journals: *Philosophy of Science*. On the basis of a pre-existing topic modeling, we develop a methodology with which we infer a set of 96 topic associative rules that characterize specific types of articles depending on how these articles combine topics in peculiar patterns. Such rules offer a finer-grained window onto the semantic content of the corpus and can be interpreted as “topical recipes” for distinct types of philosophy of science articles. Examining rule networks and rule predictive success for different article types, we find a positive correlation between topological features of rule networks (connectivity) and the reliability of rule predictions (as summarized by the F-measure). Topic associative rules thereby not only contribute to characterizing the semantic contents of corpora at a finer granularity than topic modeling, but may also help to classify documents or identify document types, for instance to improve natural language generation processes.

## Introduction

Scientific articles have semantic characteristics that are usually quite specific to their disciplinary origins. The semantic content of, say, articles in biology or sociology usually differs quite strongly from the semantic content of articles in history or philosophy (though overlap will no doubt exist on the margins, for instance in the case of articles on the history of sociology or the philosophy of the historical sciences). After all, disciplines target specific subject matters, as do sub-disciplines or even specific articles within given journals. As a consequence, analyses of large sets of scientific texts should reveal semantic features that closely track research interests. Text-mining methodologies offer a broad range of insights about such features and appropriately complement exegetic or expert-based analyses as can be found in review articles, encyclopedia entries, handbooks as well as in textbooks, anthologies or historical pieces. Topic-modeling algorithms in particular make it possible to identify “bags of words” or “topics” running throughout corpora and have been shown to be extremely effective at revealing the thematic content of documents. Such approaches, however, remain limited when it comes to investigating at a finer grained level the extent to which topics are jointly used together in specific documents and form particular associative patterns. Our objective here is to propose an approach to identify such associative patterns in large corpora of scientific articles and offer ways to characterize them. We develop a rule-based approach that builds onto topic-modeling and makes it possible to uncover how topics are associated together within given sets of documents, following what can be called “topic associative rules”. As a case study, we used a corpus from a particular subdiscipline of the humanities, the philosophy of science. This corpus consists of the complete full-text collection of all research articles published in the journal *Philosophy of Science*, one of the major journals of the field, from 1934 until 2015. Our objective was not only to assess the extent to which articles would form specific clusters based on their semantic similarity in terms of topical content but also to identify the semantic drivers for these clusters and investigate their relationships. To this aim, we devised an approach that takes as input the topics of a topic-modeling analysis and searches for associative rules between these topics. As a starting point, we used the LDA topic probability distributions over the corpus articles as analyzed in [1]. This included a list of 126 interpreted topics, with their probability distributions over the 4 602 articles of the corpus. In parallel, we carried out a k-means clustering of all the corpus articles on the basis of their word content. This clustering can be understood as a means for segregating the corpus articles into meaningful “article types”. We then searched for topic associative rules that would best characterize articles from each cluster. Associative rule mining methods have been used in many different contexts, from the identification of consumer products frequently bought together to the investigation of similarities between gene sets in biology [2–5]. Here, we applied these methods to examine associative patterns between topics. We did this with an implementation of the APRIORI algorithm [6, 7]. This approach resulted in a 17-cluster model of the *Philosophy of Science* corpus that can be characterized not just by the predominance of specific topics, but also by a set of 96 major topic associative rules. These rules show how specific topics need to be jointly present within articles for these articles to constitute exemplary representatives of given clusters. In a way, topic associative rules can be interpreted as “topical recipes” for generating philosophy of science articles of either one of the 17 cluster types. Insights on the relative topical isolation of article clusters and on the interconnectedness of topics for nearby clusters can be gained by analyzing the topology of the overall network through which rules connect topics to clusters. In addition, an analysis of rule efficacy at cluster level and rule network connectivity highlighted a positive correlation between the aggregated F-measure and cluster connectivity. Overall such analyses offer a finer-grained characterization of the semantic content of a corpus by adding a topic associative layer on top of regular topic modeling work, making salient meaningful associations of topics in subsets of documents (in the present case, offering insights about how philosophy of science topics get combined into specific types of research articles) and providing information about the relative degree of semantic complexity of each cluster. Furthermore, the correlation between connectivity and F-measure at cluster level suggests ways to identify sets of better performing rules on the sole basis of the topological properties of the cluster-level rule networks. Beyond journal-specific results, the methodology we implemented shows the type of structures that can be investigated in large corpora so as to gain further semantic insights useful for document characterization but possibly also for document classification or even content generation. In the remaining of this section, we describe the corpus that was used and the pre-existing topic-modeling that served as a basis for the present work.

### The corpus of *Philosophy of Science* and its preprocessing

The corpus consists of all full-text articles of the journal *Philosophy of Science*, from 1934 (its first year of publication) until 2015 (latest year available at the time the digital text was downloaded). These articles include all regular articles published in the journal as well as all the proceedings from the biennial meetings of the Philosophy of Science Association (PSA). These proceedings—which were published separately from the journal from their start in 1970 until 1994, and then jointly—are peer-reviewed papers, like regular articles, only slightly shorter. Editorials and book reviews were not included. The corpus thereby consists of a total of 4 602 articles (3 730 articles and 672 proceedings), amounting to 27 544 926 word occurrences, with an average word-count of about 6 000 word occurrences per article over the whole period.

Corpus preprocessing was done in a standard way—including tokenization, spelling normalization by lemmatization and morpho-syntactic disambiguation by part-of-speech tagging—so as to encode and prepare the data in a suitable way for computational analysis. Lemmatization and tagging were done using the TreeTagger algorithm [8] together with Penn TreeBank for tagging [9]. Because not all types of words are proper candidates for expressing topics and may introduce noise (for instance: determinants, prepositions or pronouns), words were filtered out depending on their morpho-syntactic tags; rare terms were also removed for similar reasons as is common practice for topic modeling. The data preprocessing stage thereby resulted in a lexicon of 10 658 distinct words distributed among 976 263 sentences.

### Topic modeling

The objective of the topic-modeling analyses was to identify the type of research interests that philosophers of science pursue in their publications, as well as how these interests evolved from 1934 up until 2015 [1]. Topic-modeling algorithms pick out word distribution patterns that can subsequently be interpreted as topics. Assuming that words are used in texts in intentional and meaningful syntagmatic combinations and that similar word combinations tend to be used to express similar meanings, studying word cooccurrence patterns can be informative about the semantic content of any given corpus. Topic modeling algorithms make it possible to identify statistical regularities among such word cooccurrences and organize them into “bags of words” that can later be interpreted as topics [10]. Topic interpretation consists in assigning to each topic a meaningful label that captures the semantic content that the topic most probable words are supposed to convey. The documents in which topics are the most probable can also be retrieved to assist in this interpretation.

To conduct the topic modeling of *Philosophy of Science*, a well-established topic-modeling algorithm based on the Latent Dirichlet Allocation model (LDA) [11] was used together with a Gibbs sampling method to facilitate convergence as described in [12] (https://pythonhosted.org/lda/api.html). While the initial number of topics in the model was set to 200 (following several runs of trial-and-error and expert judgment over topic interpretability), only 126 topics were found to be actually depicting philosophy of science research questions: 27 topics related to editorial noise (remaining html code; terms such as “note”, “section”, “figure” and so on that had escaped the preprocessing stage) and were set apart as well as 47 topics that appeared to gather generic terms of the sort often used to dress up or contextualize ideas (terms such as “question”, “answer”, “claim”, “find”, “argument”, “analysis” and numerous others that could not be interpreted as capturing any specific philosophy of science research question). It is these 126 meaningful topics and their probability distributions as previously analyzed in [1] that we took as starting point for the present study. Table 1 includes topic examples, together with their top-terms, thereby illustrating the diversity of research themes that are found in the philosophy of science, from general questions about causation and confirmation, explanation and realism, to more specific issues pertaining to the philosophy of physics, the philosophy of biology or the philosophy of mind, to name a few.

**Table 1:** Examples of topics and their top-terms.

Topic label	Top-20 terms	Topic ID
Causal-relation	causal; relation; causation; relationship; connection; probabilistic; dependence; causality; chain; asymmetry; counterfactual; analysis; lewis; relevance; account; notion; claim; intervention; event; hold	38
Confirmation	hypothesis; evidence; confirm; confirmation; instance; consequence; auxiliary; observation; glymour; test; entail; degree; report; relevance; irrelevant; support; positive; conjunction; bayesian; background	107
DN-Explanation	explanation; hempel; explain; law; model; explanandum; statistical; explanans; explanatory; salmon; account; scientific; deductive; cover; probabilistic; provide; require; event; generalization; particular	135
Evolutionary-games	strategy; game; signal; player; equilibrium; dynamics; payoff; play; stable; dynamic; population; sender; receiver; demand; round; s; skyrms; cooperation; equilibria; send	6
Genetics	gene; cell; sequence; dna; protein; code; genetic; acid; molecular; molecule; structure; product; genome; enzyme; base; organism; development; site; synthesis; expression	194
Perception	experience; perception; object; sense; consciousness; perceive; pain; sensation; perceptual; immediate; phenomenal; direct; report; subject; conscious; thing; awareness; mind; quality; subjective	199
Population-genetics	population; gene; frequency; genetic; fitness; generation; genotype; allele; mutation; selection; variance; change; variation; locus; effect; size; phenotype; trait; genetics; phenotypic	173
Psychology-cognitive	psychology; cognitive; psychological; mental; state; content; fodor; mind; intentional; computational; argue; folk; representation; cognition; representational; process; perceptual; belief; psychologist; stich	195
Quantum-mechanics	quantum; mechanic; classical; theory; mechanical; statistical; interpretation; physic; bohr; newtonian; particle; measurement; relativistic; formalism; heisenberg; field; formulation; bohm; standard; collapse	8
Rational-choice	decision; utility; preference; expect; choice; rational; option; agent; choose; act; maximize; make; prefer; action; rule; consequence; problem; theory; outcome; optimal	168
Realism	realism; realist; truth; scientific; theory; claim; success; position; argument; commitment; putnam; approximate; argue; view; ontological; empiricist; attitude; fine; theoretical; constructive	4
Relativity	frame; clock; light; velocity; time; reference; relative; absolute; move; simultaneity; observer; rest; speed; inertial; motion; rod; signal; transport; einstein; standard	19
Sentence-predicate	sentence; predicate; contain; form; language; formula; express; constant; primitive; universal; quantifier; expression; singular; equivalent; occur; statement; basic; follow; imperative; ramsey	140
Social-science	social; science; culture; society; study; cultural; human; historical; history; political; sociology; institution; anthropology; sociological; man; life; practice; anthropologist; natural; people	22
Space-geometry	spacetime; point; space-time; field; structure; metric; space; manifold; local; curve; region; geometry; vector; global; define; minkowski; coordinate; curvature; connection; tensor	41
Species	specie; species; organism; biological; taxon; individual; concept; lineage; evolutionary; common; group; classification; population; member; biologist; hull; phylogenetic; ancestor; category; taxonomy	93
Thermodynamics-Entropy	system; entropy; state; statistical; equilibrium; phase; macroscopic; ensemble; transition; time; distribution; initial; thermodynamic; microscopic; approach; ergodic; thermodynamics; demon; macrostate; evolve	55

Sample of topics, with their label, their 20 most probable words (sorted in decreasing order), and their ID number. To make this table more meaningful, we show the topics that were the most probable following the clustering analyses explained below (see Fig 3).

### Methodology

Starting from the topics and their probability distributions over the corpus articles, our objective was to investigate whether topics could be used as a basis to construct topic-associative rules that would be informative of the type of research found in *Philosophy of Science*. Studying these rules should indeed provide insights on the semantic structure of the corpus and its topology, and offer ways to analyze how specific topics need to be associated together to characterize well-defined types of articles. So to speak, we set ourselves to identify the “topical recipes” of *Philosophy of Science* articles. To do so, we followed a three-stage approach. First, we identified the different broad types of articles present in the journal by implementing a clustering analysis (on the basis of the topics present in each article). Second, we implemented a rule search algorithm to infer a (quite) large set of associative rules taking topics as antecedents and leading to any one of the specific clusters previously identified. We then evaluated all these rules on the basis of their performance as captured by their F-measure. This last step resulted in 96 rules which we then analyzed in detail, as well as the topology of their network. We describe these three methodological stages in what follows.

### Identification of *Philosophy of Science* article clusters

Since our aim was to analyze the semantic content of the journal, a first step was to investigate the extent to which its articles could be grouped into meaningful clusters—or “article types”—on the basis of their content. A very effective way to model textual content is by means of topic modeling analyses. We therefore took, as input for the clustering, the topic probability distributions that was obtained in the previous study of Malaterre *et al*. [1]. Our approach therefore was to analyze the clustering patterns that *Philosophy of Science* articles formed on the basis of their content similarity as expressed by the 126 topics that had been previously interpreted. To do so, an article vector space was generated with all articles and their normalized topic probability distributions and we implemented a k-means clustering analysis applied to articles [13]. To estimate an appropriate number *k* of clusters, we used, as heuristics, the results from 2D factorized representations of the article vector space using the t-Distributed Stochastic Neighbor Embedding (t-SNE) algorithm [14], as well as trial-and-error runs and manual analyses of cluster content (supported by our own expert-judgment of the field). On that basis, we ended up estimating that a good *k* value candidate would be *k* = 17. We therefore settled on a k-means partitioning of all the 4602 articles into 17 clusters (meaning that every article got assigned to exactly one cluster).

### Induction of topic associative rules

Our objective was to identify which topic associative rules were present in the corpus, such rules describing the combinations that topics form for articles belonging to the same clusters. Ideally, there should be enough rules to cover each cluster but not too many so as to remain qualitatively interpretable. Hence a two-step approach that consisted in, first, generating a very high number of rules and then filtering these rules to retain the best performing ones (as explained below). In order to focus on the most significant topics per article and reduce statistical noise in the topics probability distributions, we applied an elbow-based dichotomization technique to the topic probability distribution matrix [15]. This operation resulted in an *article* x *topic* matrix Δ with binary topic presence/absence for each article. In this matrix, each article exhibits on average about 11 topics, and each topic can be found in some 396 articles. We then implemented an unsupervised machine learning algorithm for rule induction that took this matrix Δ as input. We used the APRIORI approach [6, 16] (https://cran.r-project.org/web/packages/arules/index.html). In this context, associative rules are *if then* rules that take as antecedents a conjunction of topics and as consequent any one of the 17 article clusters. Such rules can be interpreted as specifying the sets of topics that an article should exhibit in order to belong to a specific cluster. Associative rules are characterized by a number of properties. Of particular importance is rule *support* which characterizes the proportion of cases for which the rule antecedents are found to be true (independently of whether the consequent is also true or not). In the present case, the support of any given rule is the proportion of articles in the corpus for which the topics specified by the rule are indeed present in the article (independently of whether the article cluster predicted by the rule is true or not). Another property that rules may exhibit is *maximality*. Maximality can be defined as the property of having the most inclusive set of antecedents. A noteworthy characteristic of maximality is to drastically reduce the number of rules without compromising their predictive accuracy. In the present study, when inferring the topic associative rules, we set a minimal support threshold of 0.004 in order to keep only relatively frequent rules (this corresponded to rules whose antecedents applied to at least 0.4% of the corpus, i.e. 18 articles), and we only retained maximal rules (which are the most informative, since they include the largest number of antecedents). This resulted in a total of 6 875 rules. While such a number of rules covered all 17 clusters, it appeared too high to be qualitatively informative about the corpus. We therefore investigated whether it could be reduced without too much loss. Hence the following third methodological step.

### Identification of most significant rules

**Fig 1:**
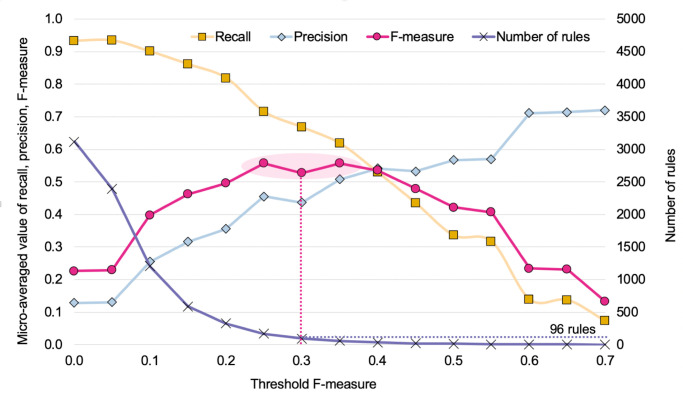
Choice of threshold F-measure. Micro-averaged recall, precision and F-measure (left-side *y*-axis) as well as number of rules (right-side *y*-axis) for sets of rules above given threshold values (*x*-axis).

Several coefficients exist for evaluating rules predictive accuracy, and on this basis select subsets of rules that best describe the associative patterns of topics and the clustering of *Philosophy of Science* articles that are observed. In the context of bimodal rules—that is to say, rules, with antecedents (article topics) that are of a different nature compared to the consequents (article clusters)—rule evaluations are usually based on precision, recall and F-measure. In short, precision is the probability of a rule to correctly predict cluster assignment on the basis of its antecedents; recall is the probability of a rule to accurately retrieve all articles in a cluster from its antecedents; and the F-measure is the harmonic average of the two. In order to only keep the smallest set of maximal rules with the best global predictive accuracy, we tested the overall predictive accuracy of sets of rules above different F-measure thresholds. In other words, for different values *v* of F-measure, we only retained the maximal rules whose F-measures were greater than *v* and we calculated the micro-averaged F-measure, recall and precision of these retained rules at *v*. As can be seen in Fig 1, when the threshold value for keeping rules increases, overall precision increases as well, while overall recall decreases, which was to be expected since there are fewer and fewer rules of lesser predictive. Meanwhile, the overall F-measure has a bell shape, with a maximum value for thresholds between 0.2 and 0.4. This led us to choose a threshold value of 0.3 corresponding to a set of 96 rules. These 96 rules happened to be high enough to cover all 17 clusters while remaining low enough to allow qualitative interpretation.

## Results

### Clustering of *Philosophy of Science* articles

**Fig 2:**
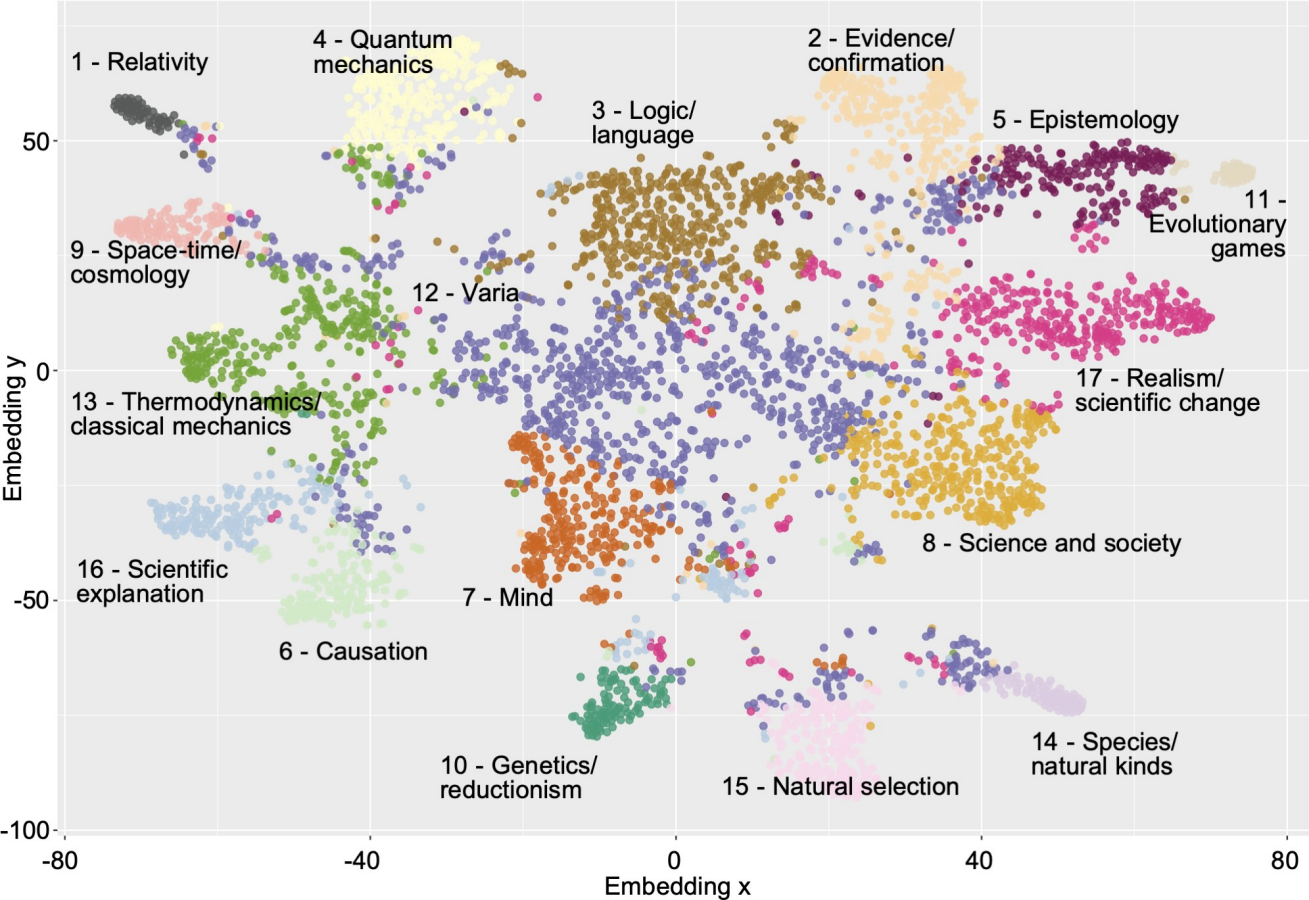
Article clusters resulting from the k-means analyses (with k = 17). Article clusters based on topic similarity (dots of the same color represent articles that belong to the same cluster; 2D factorized representation with t-Distributed Stochastic Neighbor Embedding).

**Fig 3:**
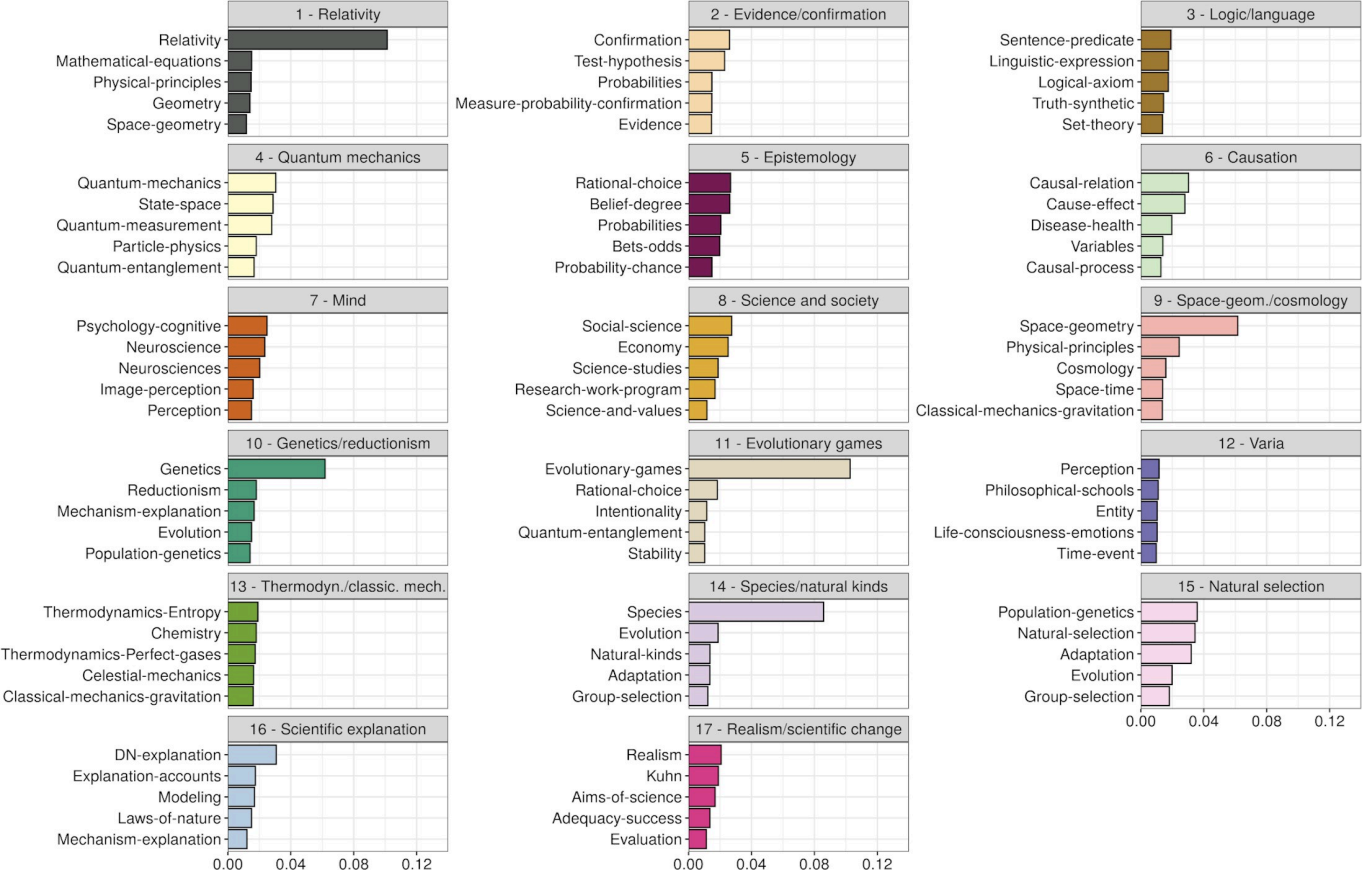
Top-5 topics per cluster. For each cluster, the 5 most probable topics are listed, while their probability of occurrence in the articles of the cluster are represented on the x-axis. Colors depict clusters (similarly to colors in Fig 2).

The k-means clustering analyses resulted in the 17 article clusters represented in Fig 2. The 17 clusters can be analyzed in terms of the average topic distribution of their respective articles (Fig 3), as well as by examining their most representative articles (S1 Table). Cluster 1 is clearly related to the philosophy of physics: it mostly concerns the theory of relativity, its mathematical treatment and its implications in terms of space geometry; notably with papers on simultaneity and isotropy, it appears most centered on philosophical questions related to special relativity. Questions about space geometry also strongly characterizes Cluster 9, in addition to space-time, gravitation and cosmology. By contrast to Cluster 1, Cluster 9 focuses more on general relativity and includes articles on space-time structure, gravitation and cosmology. Still in the domain of the philosophy of physics, Cluster 4 concerns quantum mechanics and particle physics, with papers on entanglement and decoherence, the Einstein-Podolsky-Rosen paradox and quantum measurement. Cluster 13 relates to thermodynamics, classical mechanics as well as chemistry. Representative articles include discussions about energy and its conservation, statistical mechanics, or the relation being thermodynamics and chemistry. The topical proximity of these four physics related-clusters can be seen in Fig 2, with all four clusters in the upper-left hand quadrant of the graph. On that same figure, the philosophy of biology can be spotted at the bottom of the graph with three specific clusters: Cluster 10 is about genetics as well as explanation and reductionism (the latter probably explaining the presence of the topic of evolution and the question of its reduction by molecular biology); Cluster 14 concerns the species problem, notably in relationship to natural kinds, but also with articles on pluralism and essentialism; finally, Cluster 15 is more centered on evolution by natural selection, with topics and papers on population genetics, the problem of the units and levels of selection or the interpretation of fitness, among others. Somehow also linked to biology is Cluster 11, which includes articles about evolutionary games, the emergence of cooperation, rational choice but also signaling and information (the cluster is a bit mixed, with probabilities and quantum entanglement, likely due to articles that mobilize information-related concepts). The philosophy of mind is found in Cluster 7, with articles on cognition and perception—including neuro-imaging and simulation—but also on psychology, emotions, intentionality and mental states more generally. Cluster 5 concerns epistemology, with such topics as rational choice, belief degree and probabilities, and with representative articles that concern rationality, decision theory or Bayesianism, among others. Logic and philosophy of language are found in Cluster 3, with topics about formal matters such as truth, axioms or linguistics, as exemplified also by some of its most representative articles that concern the principles of logic and logical systems. A set of four clusters somehow covers what could be termed “general philosophy of science”: Cluster 16 concerns scientific explanation and related notions such as models and laws of nature; Cluster 17 concerns realism and scientific change, including articles about the context of discovery, scientific progress, naturalism and the aims of science; Cluster 2 is about evidence and confirmation, with articles that concern for instance Hempel’s paradox, Duhem’s problem but also Bayesianism (hence a slight overlap with Cluster 5, about epistemology); lastly, Cluster 6 concerns causation, with articles on general causation, cause-effect relationships, their robustness and directionality, but also causal capacities and causal variables among others (which may also explain the presence of a topic about disease and health). Articles that have a more societal focus are found in Cluster 8, with topics such as economics, science studies and values. Finally, Cluster 12 is a more heterogeneous cluster with articles about different philosophical schools and authors (such as Whitehead, James or Singer) and broad topics that range from nature and life to mind and time. Among these articles, one finds articles published in the first half of the 20^th^ century, which are representative of a broader construal of the philosophy of science compared to what it has become now. The relative heterogeneity of this cluster is visible in Fig 2: the cluster spreads across the article space, likely collecting articles that would also not clearly fit in any other cluster.

At this stage, the clustering analyses thus reveal that *Philosophy of Science* articles can be grouped in a number of relatively homogeneous clusters on the basis of their topical content. The lists of the most probable topics in these articles give general indications of the research themes that are investigated in the articles of each cluster. Yet how these topics are more precisely found in specific associations with one another remains to be investigated. This what associative rules are about.

### The 96 topic-associative rules and their network

**Fig 4:**
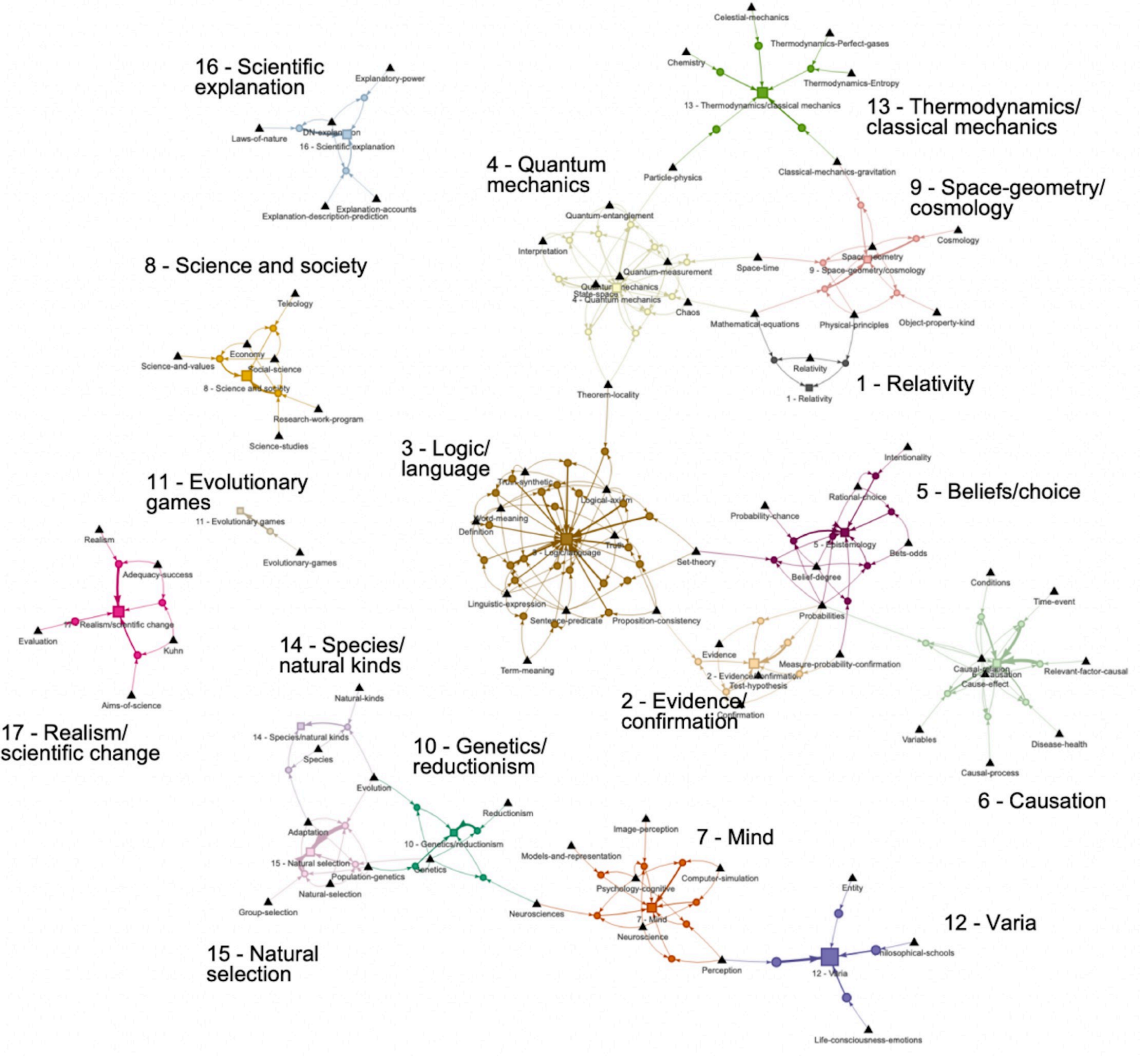
Network representation of the 96 associative rules. Each rule (circle) goes from topics (triangles) to article clusters (squares) (arrow thickness proportional to rule F-measure; size of squares proportional to the number of articles predicted in each cluster) (available online: https://chairephilosciences.uqam.ca/the-recipes-of-philosophy-of-science-characterizing-the-semantic-structure-of-corpora-by-means-of-topic-associative-rules/).

All 96 topic-associative rules that resulted from our analyses are depicted in Fig 4. Such rules make it possible to predict, for any article in the corpus, its most likely cluster on the basis of the topics that are known to be present in that article. For instance, the joint presence of the topics Laws of nature and DN-explanation in an article is a strong predictor of that article being in Cluster 16. Metaphorically speaking, topic associative rules are like “recipes” for specific types of articles: the most significant terms of the topics Laws of nature and DN-explanation can be pictured as key ingredients for producing an article of the Cluster 16 type. Note in that case that the recipe is not unique: two other topic-associative rules may be used as predictors to that same cluster, one rule that associates the topics DN-explanation and Explanatory-power, and another that associates DN-explanation, Explanation-account and Explanation-description-prediction.

Examining the topology of the rule network is one way of characterizing the semantic structure that is present within the corpus: whereas some clusters of articles have few rules based on a few very specific topics (such as cluster 11 on evolutionary games or cluster 16 on scientific explanation), others have numerous interconnected rules that involve many shared topics between rules (for instance, cluster 3 on philosophy of logic and language). We distinguish three levels of analysis.

From a macro-level perspective, the topology of the 96 rules reveals three main groups of rules that are specific in terms of their connectivity. A first group includes a set of 8 interconnected clusters that harvest the most numerous and densely connected sets of rules and that are themselves interconnected (central and upper right-hand quadrant of Fig 4). These rules concern some of the more general and historically central topics in the philosophy of science, such as philosophy of logic and language (cluster 3), epistemology (cluster 5), evidence and confirmation (cluster 2), causation (cluster 6), as well as topics in the philosophy of physics, be they about quantum mechanics, relativity, cosmology, classical mechanics or thermodynamics (clusters 1, 4, 9, 13). A second group of interconnected rules mainly concerns biology-related topics about natural selection, species, genetics and reductionism (clusters 15, 14, 10) as well as philosophy of mind topics and varia (clusters 7 and 12) (bottom part of Fig 4). Finally, a third group is apparent in Fig 4 that includes four isolated sets of rules that appear to concern well-delineated and specific topics about scientific explanation, science and society, evolutionary games and realism/scientific change (clusters 16, 8, 11, and 17).

At a mezzo-level, each one of these three large groupings of rules can be analyzed in terms of how clusters connect to one another. For instance, cluster 3 (philosophy of logic and language) includes rules that connect to three other clusters (clusters 2, 4 and 5, respectively about evidence/confirmation, quantum mechanics and epistemology) through three shared topics: Measure-probability-confirmation, Set-theory and Theorem-locality. On the other hand, cluster 6 (causation) connects to two clusters through only one shared topic Probabilities: cluster 2 (evidence/confirmation) and cluster 5 (epistemology).

**Fig 5:**
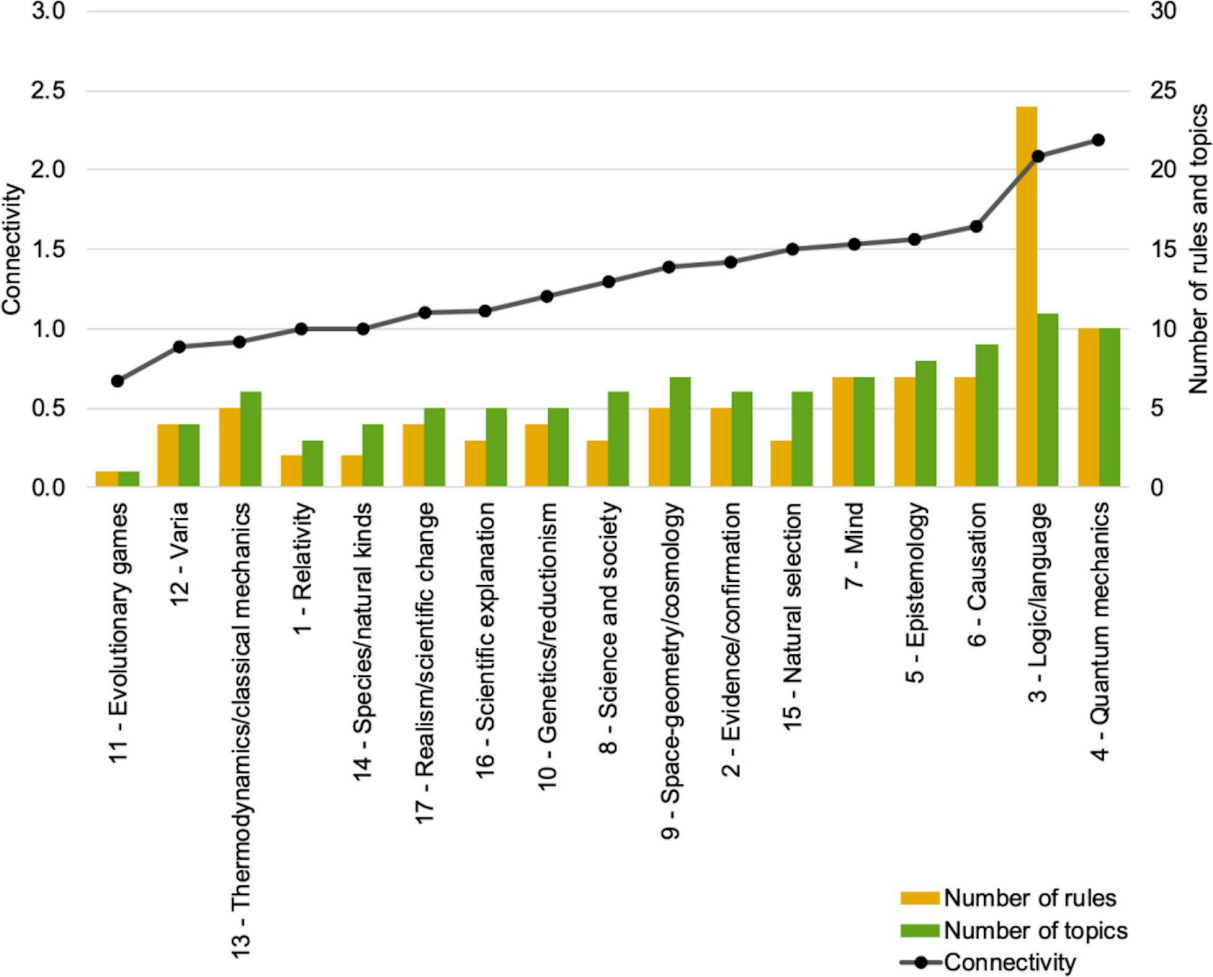
Cluster network connectivity, rules and topics. Rule network connectivity (defined as the ratio of the number of edges (arrows linking topics to rules and rules to clusters as in Fig 4) divided by the number of nodes (topics, rules, cluster) for each cluster; left-side *y*-axis) per cluster (*x*-axis), sorted by increasing connectivity. Number of rules and topics are also represented (right-side *y*-axis).

Finally, the topology of the 96 rules can be characterized at a micro-level perspective by specifically examining the associative rules of a given cluster, their relative significance and their interconnections. For instance, cluster 3 (philosophy of logic and language) is characterized by a densely connected set of 24 rules that take as antecedents some 11 topics. On the other hand, cluster 11 (evolutionary games) only has one rule based on a single topic. Such characteristics can be used to compute a connectivity measure at cluster level that reveals how dense the rule networks are depending on clusters (Fig 5).

### Applying topic-associative rules to characterize documents

**Fig 6:**
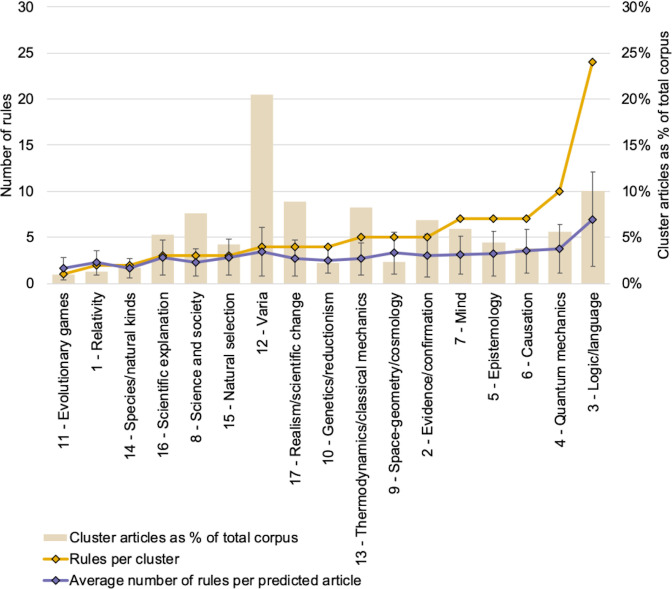
Rules per cluster. For each cluster (*x*-axis), number of rules pointing to that cluster as well as average number of rules per correctly predicted article and standard deviation (left-side *y*-axis); relative cluster sizes as % of total number of corpus articles represented in the background (right-side *y*-axis); clusters sorted by increasing number of rules.

By construction, topic-associative rules predict an article type (a cluster) depending on the presence of specific topics. Given a particular article and its distribution of topics, rules may or not apply, and may or not predict its correct cluster. Remember that the set of 96 rules was chosen as a compromise between overall precision and recall. As can be seen on Fig 6, the 96 rules are not evenly spread across clusters, meaning that not all documents in the corpus can be similarly characterized by means of these rules. Out of the total number of articles in the cluster, about 85% are such that at least one topic associative rule applies. In practice, it is often the case that many rules apply to each one of these 85% articles, in some cases making erroneous cluster predictions (43.6%) (as illustration, Table 2 lists the rules that apply to two random articles in the corpus). On average, every article for which a prediction can be made receives 3.5 rule applications. As can be seen on Fig 6, this average varies per cluster, from slightly less than 2 for cluster 11 (on evolutionary games) up to 7 for cluster 3 (logic/language).

**Table 2:** Examples of topic associative rules.

Article ID	Rule ID	Rule antecedents (topics)	Predicted article cluster	Actual article cluster	Rule prediction status
3786	377	Chemistry	13	4	False
3786	417	Particle-physics	13	4	False
3786	3859	Interpretation AND Quantum-entanglement AND State-space	4	4	True
3786	3862	Quantum-mechanics AND Interpretation AND State-space	4	4	True
3786	5867	Quantum-mechanics AND Interpretation AND Quantum-measurement AND Quantum-entanglement	4	4	True
3786	5898	Quantum-mechanics AND Quantum-measurement AND Chaos AND State-space	4	4	True
3786	5920	Quantum-mechanics AND Quantum-measurement AND Chaos AND Quantum-entanglement	4	4	True
3786	6650	Quantum-mechanics AND Particle-physics AND Quantum-measurement AND Quantum-entanglement AND State-space	4	4	True
1704	3073	Sentence-predicate AND Set-theory	3	16	False
1704	394	Classical-mechanics-gravitation	13	16	False
1704	947	DN-explanation AND Explanatory-power	16	16	True
1704	961	Laws-of-nature AND DN-explanation	16	16	True
1704	3743	DN-explanation AND Explanation-accounts AND Explanation-description-prediction	16	16	True

List of rules that apply to two articles: one from cluster 4 about quantum mechanics (article 3786: Cartwright, Nancy (1972) “A Dilemma for the Traditional Interpretation of Quantum Mixtures”) and the other from cluster 16 about scientific explanation (article 1704: Kitcher, Philip (1981) “Explanatory Unification”). Rules have a number of topics as antecedents (to be found in the article) and point to a predicted cluster topic (for that article). Comparison of predicted versus actual article cluster results in a true/false rule prediction status.

**Fig 7:**
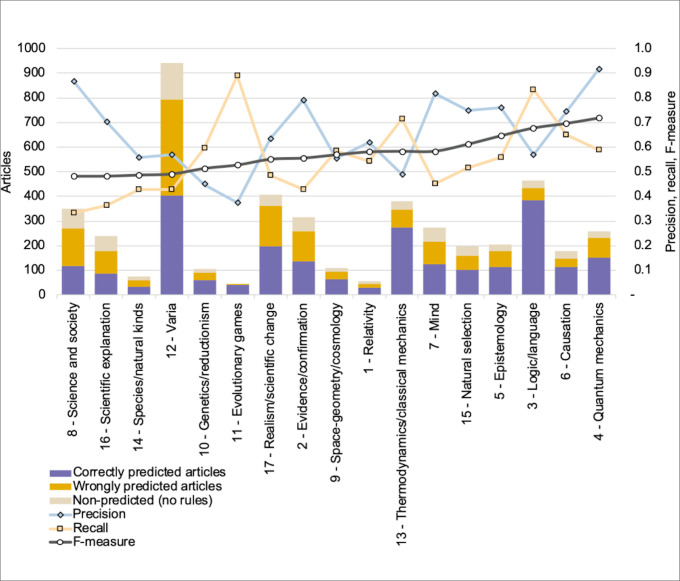
Precision, recall and F-measure per cluster. For each cluster (*x*-axis), average precision, recall and F-measure (right-side *y*-axis) as well as number of articles split into three types: correctly predicted articles, wrongly predicted articles and non-predicted articles (left-side *y*-axis); clusters sorted by increasing F-measure.

Using a majority rule for each article, we retained as predicted-cluster the cluster that had been the most often predicted by the rules applying to that article (rule “votes” being weighted by rule F-measure). We thereby calculated the number of articles of a given cluster that had been rightly predicted; we also assessed the number of articles from other clusters that had been erroneously attributed to that cluster as well as the number of articles from the cluster that were not predicted to belong to that cluster. We thereby calculated, at cluster level, the average precision and recall for all the 96 rules, as shown on Fig 7. Precision fluctuates between 37% and 88%, and recall between 27% and 87%. As can be expected, clusters with higher scores on precision tend to have lower scores on recall, yet results vary strongly depending on clusters. When sorted based on their F-measure, a set of five clusters have scores higher than 60%: clusters 4, 6, 3, 5 and 15 that respectively concern quantum mechanics, causation, philosophy of logic and language, epistemology and natural selection. In these five cases, both precision and recall are above 50%. Four of these clusters are the ones in which the number of rules is the highest (between 7 and 25). Note in particular for cluster 3 (philosophy of language and logic) how the number of rules makes up for low precision by increasing recall. At the other end of the spectrum, four clusters have an F-measure as well as a recall below 50%: clusters 8, 12, 14, 16 that concern science and society, varia, species/natural kinds and scientific explanation. Such results could have been expected for the varia cluster, which gathers a relatively large number of diverse articles (20% of all articles); they also make sense for the clusters about science and society and species/natural kinds which tend to be somehow heterogeneous clusters mixing related yet distinct types of articles; a similar phenomenon may be at work for the cluster about scientific explanation. Two clusters are notably characterized by high precision (above 70%) and low recall (below 40%): cluster 8 about science and society and cluster 16 about scientific explanation. Articles in these clusters thereby tend to be relatively heterogeneous (low recall) but include a subset which is easily identifiable by a few sets of rules (high precision).

### Network structure and rules success

**Fig 8:**
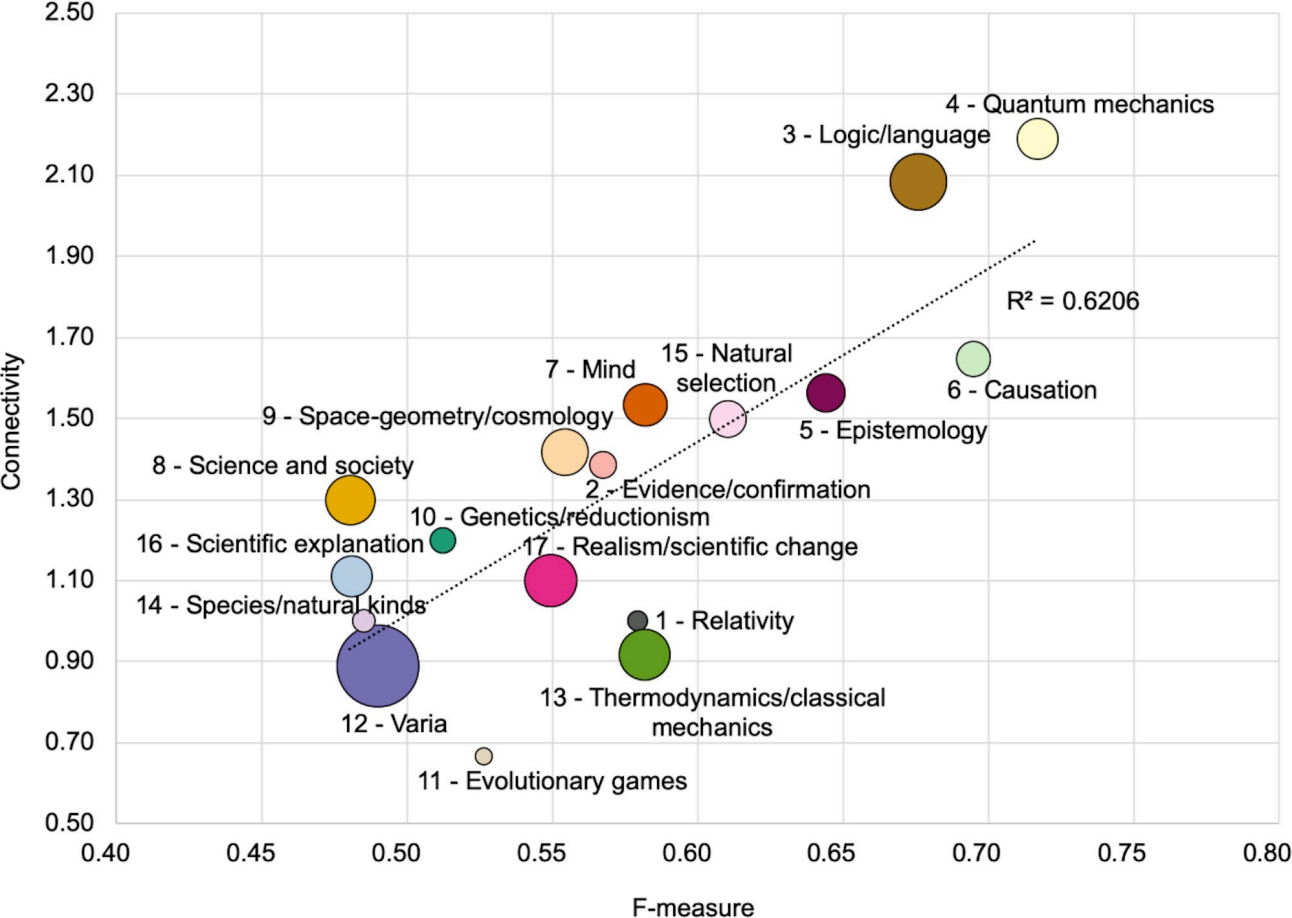
Cluster connectivity and F-measure. Position of clusters based on their respective F-measure (*x*-axis) and connectivity (*y*-axis); size proportional to number of articles per cluster.

Jointly examining connectivity and F-measure reveals a positive correlation between the two. As shown in Fig 8, the clusters that have the highest rule-network connectivity also tend to have the highest scores in F-measure, and the other way around. For instance, clusters 3, 4, 5 and 6 (respectively about philosophy of logic/language, quantum mechanics, epistemology and causation) simultaneously display the highest connectivity and F-measure compared to any other cluster. At the other end of the spectrum, clusters such as 8, 11, 12, 14, 16 (about science and society, evolutionary games, varia, species/natural kinds and scientific explanation) display both a low rule-network connectivity and a low F-measure. This indicates that a topological metric of the rules network—which can be estimated just by examining the network that rules form, i.e. without assessing any rule F-measure—can be a good indicator of rule predictive efficacy at cluster level. Such a correlation makes sense since the best performing clusters in terms of F-measure all tend to have more topics and more interconnections, and the other way around (see Fig 4). But this need not always be so: one could imagine highly effective rules based on just a few but very specific topics. In such a case, predictive efficacy would be high despite a low connectivity.

**Fig 9:**
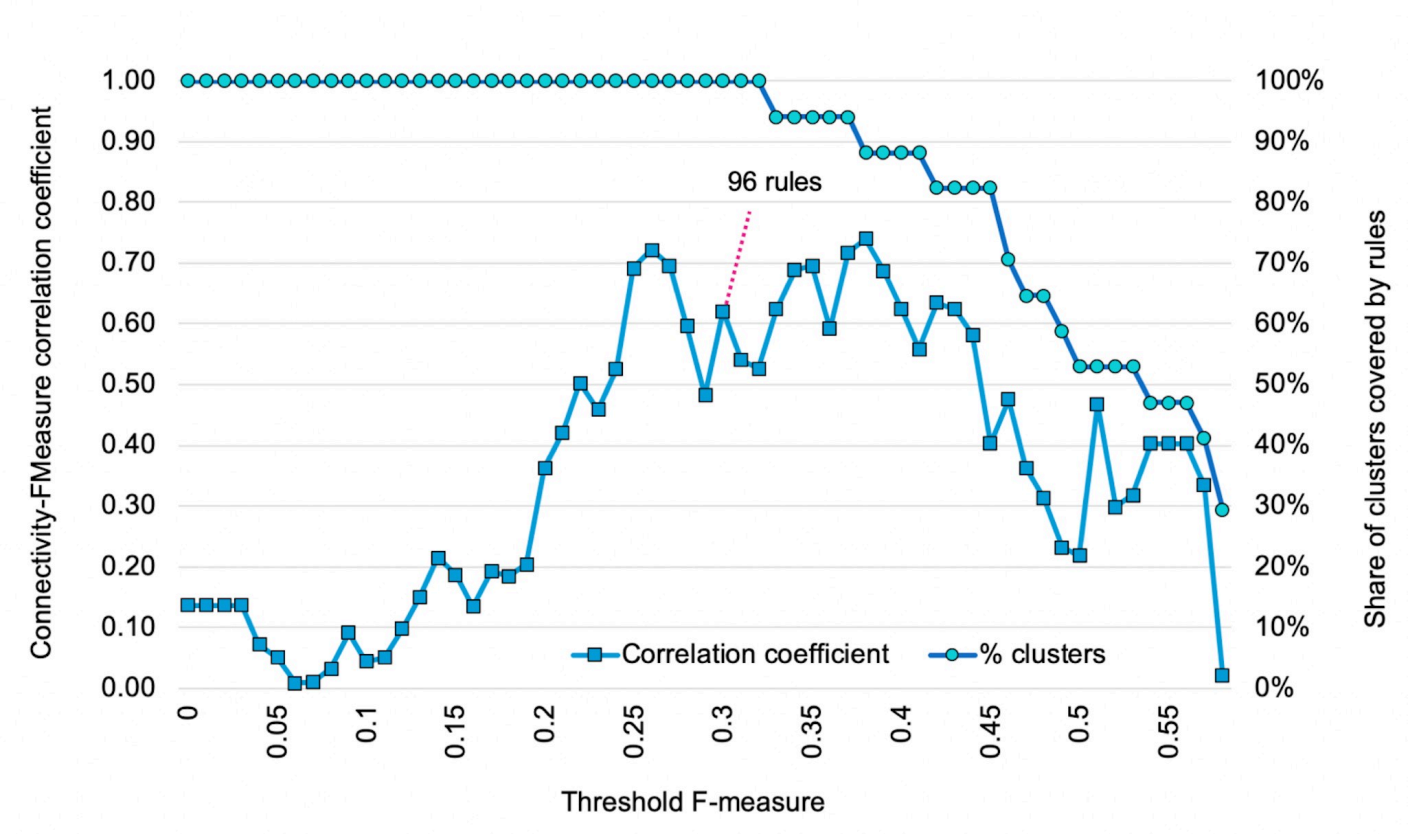
Correlation between cluster connectivity and F-measure. Correlation coefficient R^2^ between connectivity and F-measure assessed at cluster level (left-side *y*-axis) for different values of threshold F-measure (*x*-axis); relative number of clusters covered by rules, expressed in % (right-side *y*-axis).

To check for robustness, we systematically examined all rules networks obtained for different F-measure threshold values. That is to say, instead of focusing on the threshold value of 0.3 that had led to the selection of the 96 rules (middle of the bell curve on Fig 1), we considered rule networks that could be built at different F-measure threshold values. For each network, we calculated cluster connectivity and cluster-level F-measure, and assessed their correlation (Fig 9). Rules networks span from several thousand rules for very low values of threshold F-measure to about one hundred rules for a value of 0.3 and down to just a handful at values above 0.5 (as could be seen on Fig 1). Note that above 0.33, some of the 17 clusters start being absent from the rule-networks (over half of them being missing above 0.54). Among the rule networks that cover all clusters (hence below 0.33), increasing the number of rules tends to worsen the correlation coefficient between cluster connectivity and F-measure, the best compromise being around a value of 0.25 (corresponding to 169 rules). The most effective sets of rules thereby also tend to be these sets of rules that maximize cluster-level rule network connectivity.

## Discussion

Topic associative rules characterize the degree of topical interdependencies and the degree of representativeness of these interdependencies within a given cluster. As such, they provide two major insights into the semantic content of a corpus that go beyond what can be learned from topic-modeling alone: a structural insight provided by the topology that rules form over specific subsets of the corpus articles and a predictive insight given by the rules themselves and their predictive success. Since the corpus we used is a corpus in the philosophy of science, some of these insights relate to the very nature of research topics and articles in this field of the humanities, yet, other more methodological insights have a broader scope that is not domain specific.

As our results show, rules form networks over the whole corpus whose topology can be examined. By construction, rules are cluster specific (since each rule only points to a single cluster); nevertheless, rules may share topics among their antecedents. It is through these shared topics that rules form networks all throughout the corpus. A first finding concerns the fact that the ‘micro-level’ structure of these networks at the level of individual clusters varies strongly from cluster to cluster. In particular, the number of rules per cluster varies significantly from 1 to 24 rules per cluster, with an average of 5.6 rules per cluster. These variations are not correlated with the number of documents per cluster but appear to capture the topical tightness of clusters. Cluster 3 (philosophy of logic and language) is the cluster with the highest number of rules and the highest connectivity, while capturing about 10% of all corpus articles. This bears witness to the well-known centrality of philosophy of language and logic in the philosophy of science, especially in the early 20^th^ century, both from a historical and a conceptual point of view. It also shows in that numerous topics of interest were tightly interconnected in different articles, for instance about truth, meaning, definition, sentence, proposition or consistency. At the other extreme, cluster 11 is an example of a very minimalist cluster characterized by only one rule with one topic (about evolutionary games) and with the lowest connectivity of all. In that case, it is likely that the very small number of related articles—about 1% of all corpus articles—resulted in very few topics and even fewer rules. That cluster indeed characterizes a very specialized domain of investigation. On the other hand, few rules and a very low connectivity also characterize cluster 12 (varia). In that case, however, the number of articles is the highest of all clusters, reaching over 20% of all corpus articles. The reason behind the small number of topic-associative rules appears to come from the high diversity of articles in that large cluster, with many different and often unrelated topics (including wide ranging topics about life and mind, about ontology or about classical history of philosophy). Overall therefore, connectivity of rules network tends to make sense from what is known of the field and the articles in each cluster. At a ‘macro-level perspective’ as we called it, inter-cluster connections also tend to make sense, though the methods also led to some unexpected results. All clusters related to the philosophy of physics appear as interconnected (thermodynamics/classical mechanics, cosmology, relativity and quantum mechanics) through a few specific topics. Similarly, the clusters that relate to the theory of knowledge (epistemology, evidence/confirmation) are also interconnected, as well as with causation (through a topic about probability, which makes perfect sense given recent accounts of causation). The philosophy of language and logic appears as a central hub connected to both philosophy of physics and theory of knowledge. All clusters about the philosophy of biology also appear interconnected (about natural selection, species, genetics, reductionism), and connected as well to the philosophy of mind. What may seem more surprising is the connection of the cluster varia to the cluster mind (though this connection is due to a topic about perception): one would have typically expected this cluster to be connected for instance with cluster 8 (a fairly broad cluster about science and society) or with cluster 17 (about scientific realism and scientific change). Another unexpected result is also the isolation of cluster 16 about scientific explanation: this a well-known research theme in the philosophy of science, often related to issues about causation and probability, and one would have expected connections with clusters 2, 5 or 6 among others. Of course, the topological structures that are visible with a set of 96 rules will become increasingly blurred in models based on a higher number of rules (i.e. lower F-measure threshold), more and more connections being added within clusters (‘micro-level’) but also between clusters (‘mezzo-level’) and between groups of clusters (‘macro-level’). On the opposite, the topological structures will tend to vanish with models based on fewer and fewer rules (i.e. more stringent higher F-measure threshold), gradually losing clusters as well. Hence the importance of choosing a principled number of rules (see methodology section).

Besides network topology, the second insight rules can provide about a corpus is a form of predictive insight about the semantic content of specific types of articles. In the present case-study which concerns a corpus in the philosophy of science, this means that some specific types of articles in that domain of the humanities appear to be easier to predict than others. More generally, rule predictive success can be seen as a compromise between rule precision (i.e., the frequency with which rules that apply to given articles correctly predict their clusters) and recall (i.e., the extent to which all articles of a given cluster are correctly predicted to belong to that cluster), hence the use of their F-measure (harmonic average). The results show that predictive success varies significantly from cluster to cluster, with no correlation with cluster size. For instance, one of the clusters with the highest F-measure is cluster 4 (about quantum mechanics), which is about the size as cluster 16 (scientific explanation), the latter having one of the worst F-measure. As suggested above, predictive success seems to be well correlated with rules connectivity at the cluster level. Retrospectively, this makes sense in so far as the more interwoven topics are, the more distinctive their signature will be.

Of course, this relationship between rule-connectivity and rule-success would deserve further testing with other corpora and in other domains. If corroborated, such relationship would reveal that connectivity—a structural characteristic of the rules network—could be a good proxy for assessing predictive success, thereby showing that structural features of topic-associative rules may strongly contribute to characterizing article semantic content. Another question that could also be addressed concerns the evolution of the clusters and rules network over time. Here we took a synchronic viewpoint that made us consider all corpus articles at the same time. A diachronic viewpoint based on article publication years would make it possible to investigate how article clusters and associative rules networks evolved over time (for a diachronic analysis that simply concerns the evolution of topics in the philosophy of science, see also [17]).

Identifying topic-associative rules thereby makes it possible to investigate the patterns that topics form within sets of articles of specific corpora. It gives an additional viewpoint that builds on top of topic-modeling analyses and provides a finer-grained characterization of how topics are used together in specific research articles. Such a characterization should be of interest to domain specialists (in the present case, philosophers of science and historians of philosophy) as it provides perspectives that are complementary to existing exegetic and expert-based analyses of the field. Associative rules also make it possible to predict an article type on the basis of the presence or not of specific topics in an article. Such predictive capabilities may be used to improve document classification practices or, in another instance, to enhance text generation tailored to specific semantic contexts. Indeed, since topic-associative rules helps uncovering topical associative patterns—or “topical recipes”—in specific sets of texts, they not only describe how topics are used in actual documents but also provide information about how topics should be associated to generate textual content of a specific kind. Automatic generation of text—or “Natural Language Generation” (NLG)—has been developed in many areas and with many different approaches [18], from the production of texts on the basis of structured data, for instance, weather forecasts based on meteorological data [19] or financial summaries based on business data, to the creation of pseudo-journalistic articles [20], chatbot discussions [21] or even pseudo-scientific articles (for instance by the well-known software SCIgen: https://pdos.csail.mit.edu/archive/scigen/) and reviews [22]. Because topics are here modeled as probability distributions over a lexicon, topic-associative rules can be taken as defining preferential associative terminological patterns. which could in turn be used to supply preferred sets of terms for corpus-based NLG, including automatic summarization. More specifically, topic-associative rules inferred from given corpora could be used to supplement NLG lexicalization by providing preferred sets of terms to be used for generating texts that would be more finely targeted to resemble specific document types—the ‘clusters’ in our study—of the chosen corpora. Furthermore, examining beforehand the topology of topic-associative rules from a given corpus could help identify semantic domains for which text generation is more likely to be successful, typically those domains with high rule connectivity.

## Conclusions

By applying classification techniques to topics inferred from topic-modeling analyses, we have shown how topic-associative rules can be inferred from a given corpus. Whereas topic-modeling can provide a first understanding of the semantic content of documents and corpora, making it possible to group documents on the basis of topic similarity, topic-associative rules provide a finer-grained characterization by specifying the specific ways in which topics are jointly found within articles of given topical clusters. For the present study, we used a corpus of journal articles from a particular subfield of the humanities: the philosophy of science. In this respect, some of the findings of our analyses are domain specific and characterize the semantic content of research articles in this field. On the other hand, other findings are of a more general methodological scope. We have proposed a methodology to infer the specific associative patterns that topics follow within clusters of similar articles. On this corpus, we identified a set of rules that appear to reliably make it possible to predict article clusters (which can be understood as the corpus main ‘article types’) on the basis of the specific presence of certain topics within these articles. In particular, we showed that certain types of articles—here, in areas that concern philosophy of language and logic, philosophy of quantum mechanics as well as philosophy of mind, causation and epistemology—are very well characterized by their topic associative rules. Articles in these clusters appear to use a quite relatively high number of interconnected topics. It is as if article ‘recipes’ included relatively many ingredients in many overlapping ways, thereby leading to the right types of articles. On the other hand, articles that belong to clusters with low connectivity tend to rely on a few scarce rules that mobilize few interconnected topics, and, as a result, recipes fail to reliably lead to the right article types. From a more methodological perspective, our results suggest a positive correlation between rules network connectivity and predictive success (as denoted by the F-measure of cluster rules). It would be interesting to find out whether such correlation holds on other corpora. In any case, simply studying the topology of the rules network can prove fruitful when it comes to characterizing the semantic content of corpora and, specifically, how this semantic content varies from one document cluster to another. These findings could in turn find useful applications for classification or, for instance, for lexicalization in corpus-based NLG approaches.

## Supporting information

S1 Table Top-10 articles per cluster. For each cluster, list of the 10 closest articles to cluster centroid (sorted by increasing distance to the centroid).
(XLSX)

S1 Data Data sets. (XLSX)
